# Aldehyde dehydrogenase 1 (ALDH1) isoform expression and potential clinical implications in hepatocellular carcinoma

**DOI:** 10.1371/journal.pone.0182208

**Published:** 2017-08-08

**Authors:** Cheng–kun Yang, Xiang–kun Wang, Xi–wen Liao, Chuang–ye Han, Ting–dong Yu, Wei Qin, Guang–zhi Zhu, Hao Su, Long Yu, Xiao–guang Liu, Si–cong Lu, Zhi–wei Chen, Zhen Liu, Ke–tuan Huang, Zheng–tao Liu, Yu Liang, Jian–lu Huang, Kai–yin Xiao, Min–hao Peng, Cheryl Ann Winkle, Stephen J. O'Brien, Tao Peng

**Affiliations:** 1 Department of Hepatobiliary Surgery, The first Affiliated Hospital of Guangxi Medical University, Nanning, Guangxi Province, China; 2 Department of Hepatobiliary and Pancreatic Surgery, The first Affiliated Hospital of Zhengzhou University, Zhengzhou, Henan Province, China; 3 Department of Hepatobiliary Surgery, Affiliated Hospital of Guangdong Medical University, Zhanjiang, Guangdong Province, China; 4 Department of Hepatobiliary Surgery, Third Affiliated Hospital of Guangxi Medical University, Nanning, Guangxi Province, China; 5 Laboratory of Genomic Diversity, National Cancer Institute, NIH, Frederick, MD, United States of America; Chang Gung Memorial Hospital Kaohsiung Branch, TAIWAN

## Abstract

Hepatocellular carcinoma (HCC) is one of the most prevalent and life-threatening malignancies worldwide. There are few diagnostic and prognostic biomarkers and druggable targets for HCC. Aldehyde dehydrogenase 1 (ALDH1) is a marker of stem cells in a variety of cancers, but the mRNA levels and prognostic value of ALDH1 isoforms in HCC patients remain unknown. In the present study, gene ontology annotation of the ALDH1 family was performed using the Database for Annotation, Visualization and Integrated Discovery (DAVID), and the gene pathway analsis was performed using GeneMANIA software. The initial prognostic value of ALDH1 expression in 360 HCC patients was assessed using the OncoLnc database. The expression levels of ALDH1 isoforms in normal liver tissues and clinical specimens of cancer *vs*. normal control datasets were determined using the GTEx and Oncomine databases, respectively. We then analyzed the prognostic value of ALDH1 expression in 212 hepatitis B virus (HBV)–related HCC patients using the GEO database. We found that the ALDH1 isoform showed high aldehyde dehydrogenase activity. The *ALDH1A1*, *ALDH1B1*, and *ALDH1L1* genes encoded for the ALDH1 enzyme. High *ALDH1B1* expression had protective qualities in HCC patients. Moreover, HBV–related HCC patients who showed high *ALDH1L1* gene expression had a better clinical outcomes. In addition, high *ALDH1A1* expression was associated with a 57–month recurrence–free survival in HBV-related HCC patients. High *ALDH1B1* expression was protective for HCCs with multiple nodules and high serum alpha–fetoprotein (AFP) level. Furthermore, high serum AFP levels contributed to lower *ALDH1L1*. *ALDH1A1*, *ALDH1B1*, and *ALDH1L1*, all of which were considered promising diagnostic and prognostic markers as well as potential drug targets.

## Introduction

Liver cancer is the fifth most common cancer and the second leading cause of cancer–related death in males worldwide, with hepatocellular carcinoma (HCC) representing the largest proportion (70%–90%) of primary liver cancers [1, 2]. Although most HCC cases (> 80%) occur in sub–Saharan Africa or Eastern Asia, China alone accounts for more than 50% of cases worldwide [3, 4]. Although early diagnostic methods, surgical resection, liver transplantation, and ablation by radiofrequency or ethanol injection have provided considerable advancements, the 5-year survival rate ranges from 50%–70% at early disease stages, and the prognosis of HCC patients remains poor due to the high postoperative recurrence rate and metastasis [5, 6]. Thus, the elucidation of mechanisms of initiation and progression of HCC and the identification of diagnostic and prognostic markers as well as potential drug targets are critical to provide better prognosis and individualized treatments for HCC patients.

Aldehyde dehydrogenases (ALDHs) are a family of intracellular enzymes that are involved in cellular detoxification, differentiation, and drug resistance by oxidation of cellular aldehydes [7]. The major function of ALDHs is aldehyde detoxification, which serves to protect stem cells against the destructive properties of oxidative aldehydes. It has been shown that both human and murine hematopoietic and neural stem cells, as well as related progenitor cells, exhibit high ALDH activity [8–11], and that increased cell differentiation leads to decreased ALDH activity [11]. As a marker of stem cells in a variety of cancers, ALDH1 acts as a modulator for cell proliferation, stem cell differentiation, and resistance to chemotherapeutic agents [12]. Tanei et al. reported that tumors from breast cancer with ALDH1–positive cells displayed higher rates of chemotherapy resistance [13]. Furthermore, because ALDH1–positive cells exhibited tumorigenic ability with prognostic significance, the ALDH1–positive status may be involved not only in proliferation of the primary tumor but also in the formation of metastases [9]. However, of the ALDH1 isoforms (*ALDH1A1*, *ALDH1A2*, *ALDH1A3*, *ALDH1B1*, *ALDH1L1*, and *ALDH1L2*) that alone or in combination contribute to ALDH1 activity have not been determined. Previous studies [14–16] have demonstrated distinct prognostic values of ALDH1 isoforms in breast cancer, gastric cancer, and non–small cell lung cancer using an online Kaplan–Meier plotted database. Nonetheless, the prognostic value of individual ALDH1 isoforms in HCC is not yet clear. In the current study, we determined the mRNA level and prognostic value of ALDH1 isoforms in HCC patients.

## Materials and methods

This study was approved by the Ethics Committee of the First Affiliated Hospital of Guangxi Medical University. The gene ontology of the *ALDH1* was annotated using the Database for Annotation, Visualization and Integrated Discovery (DAVID) (https://david.ncifcrf.gov/), which provides resources to understand the biological functions of a large group of genes [17]. Genes’ pathway analysis was performed by a GeneMANIA Software (www.genemania.org) [18]. The prognostic value of mRNA expression of ALDH1 isoforms in HCC was initially assessed using the OncoLnc database (www.oncolnc.org). OncoLnc is a tool for interactively exploring survival correlations, and it contains survival data for 8,647 patients from 21 cancer studies performed by The Cancer Genome Atlas (TCGA), along with RNA–SEQ expression for mRNAs from TCGA cases [19]. TCGA contains microarray-based and RNA sequencing–based mRNA expression variants. The survival analyses were performed using cutoff values of median or quartile of ALDH1 family expression in 360 HCC patients. mRNA expression levels above or below the cutoff value divided the cases into high expression and low expression groups, respectively. However, since the different background and type of liver cancer was analyzed in TCGA, OncLnc was subsequently used to evaluate the initial prognostic value of ALDH1 isoforms with different cutoff values. Considering the complexity of tumorigenesis and tumor progression in HCC cases, we further analyzed the association of the ALDH1 isoforms mRNA expressions with a total of 445 hepatitis B virus (HBV)–related HCC and paramalignant samples from the Gene Expression Omnibus (GEO accession: GSE14520) using the median cutoff value. Sample mRNA expression was measured using the Affymetrix HT Human Genome U133A Array (HT_HG-U133A). To explore the relationships between the ALDH1 isoforms and clinical features, we downloaded clinical data coupled to expression data for the ALDH1 isoform mRNA expressions from OncoLnc and GEO. In GSE14520, we identified 212 HBV-related HCC patients with ontology features and clinical survival information.

The mRNA expression data for the ALDH1 isoforms in normal human tissues were obtained from the Genotype Tissue Expression (GTEx) database (www.gtexportal.org). The GTEx database provides a resource to study human gene expression and regulation and their relationships to genetic variation [20]. This release of GTEx includes genotype data from approximately 450 donors and over 9600 RNA–Seq samples across 51 tissue sites and 2 cell lines as well as adequate power to detect expression quantitative trait loci in 44 tissues. Moreover, Oncomine gene expression array datasets (www.oncomine.org) were used to analyze the mRNA levels of different ALDH1 isoforms in different cancers. Oncomine is an online cancer microarray database used to facilitate the discovery of genome-wide expression analyses [21]. In this study, we compared the clinical specimens of cancer *vs*. normal control datasets, using the Student’s *t*–test to generate a *P* value. A *P* value of < 0.01 was considered statistically significant, and fold change was defined as 2, whereas the data type was restricted to mRNA.

The correlation of *ALDH1* isoform expression was analyzed by Pearson’s correlation coefficient. Kaplan–Meier survival analysis was used to calculate survival percentages, and the differences in survival percentages were then estimated using a generalized log–rank test. A Cox proportional hazard regression model was used to calculate the hazard ratio (HR) with 95% confidence interval (CI). All statistical analyses were two–sided and were performed using SPSS version 17.0 (SPSS, Chicago, IL, USA). A *P* value of < 0.05 was considered statistically significant. The survival curves and correlation plots were depicted by GraphPad 5.01 (www.graphpad.com) and R 3.3.2 (www.r-project.org), respectively.

## Results

The DAVID gene ontology terms associated with *ALDH1* isoforms are presented in S1 Table. All numbers for the *ALDH1* isoforms were enriched in aldehyde dehydrogenase activity. In addition, the *ALDH1* isoforms also catalyzed oxidation–reduction reactions and were involved in retinol metabolism.

**Fig 1:**
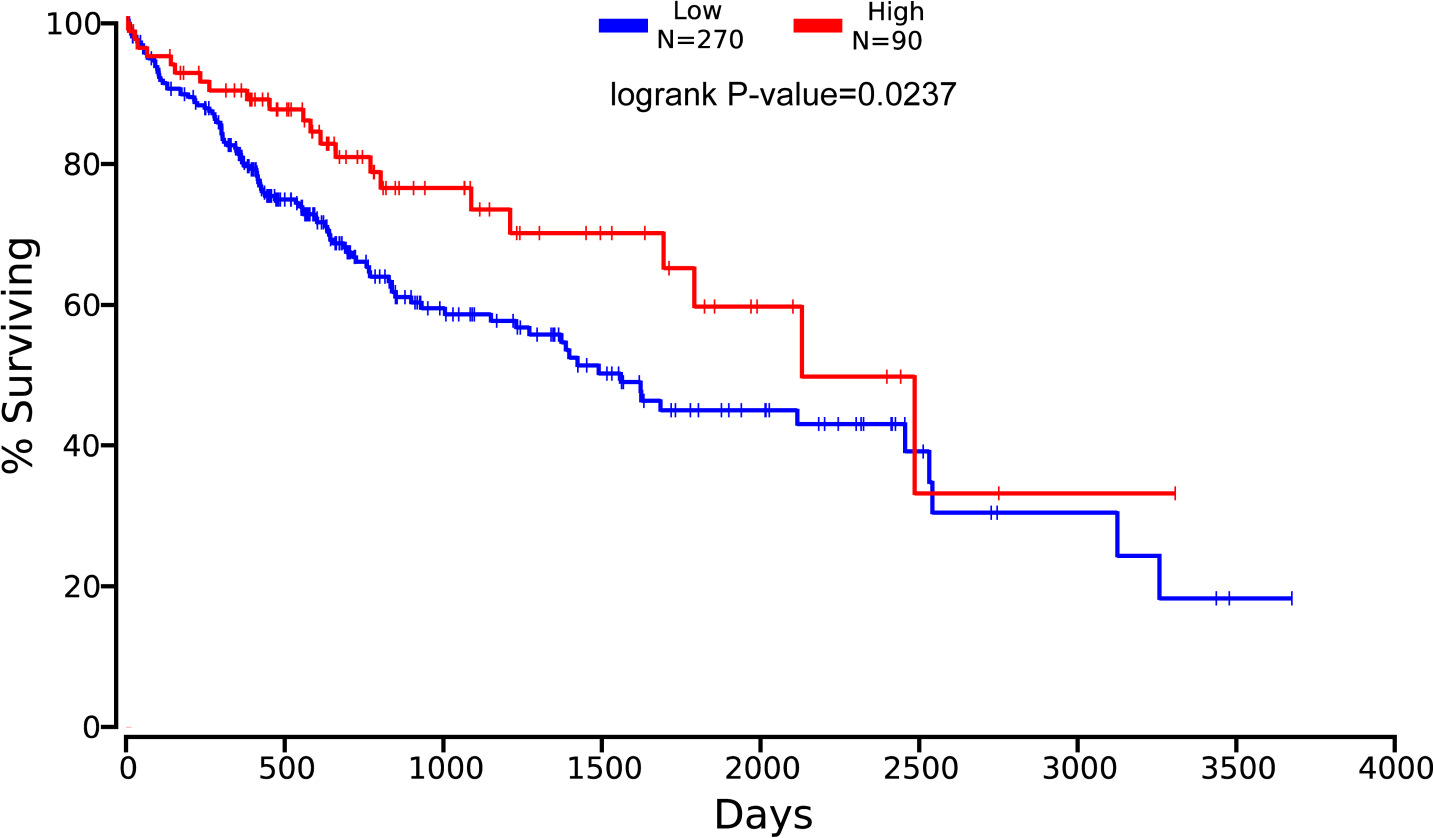
The prognostic value of *ALDH1B1* mRNA expression in liver cancer patients, and the survival curve as plotted using the OncoLnc database. ALDH = aldehyde dehydrogenase.

**Fig 2:**
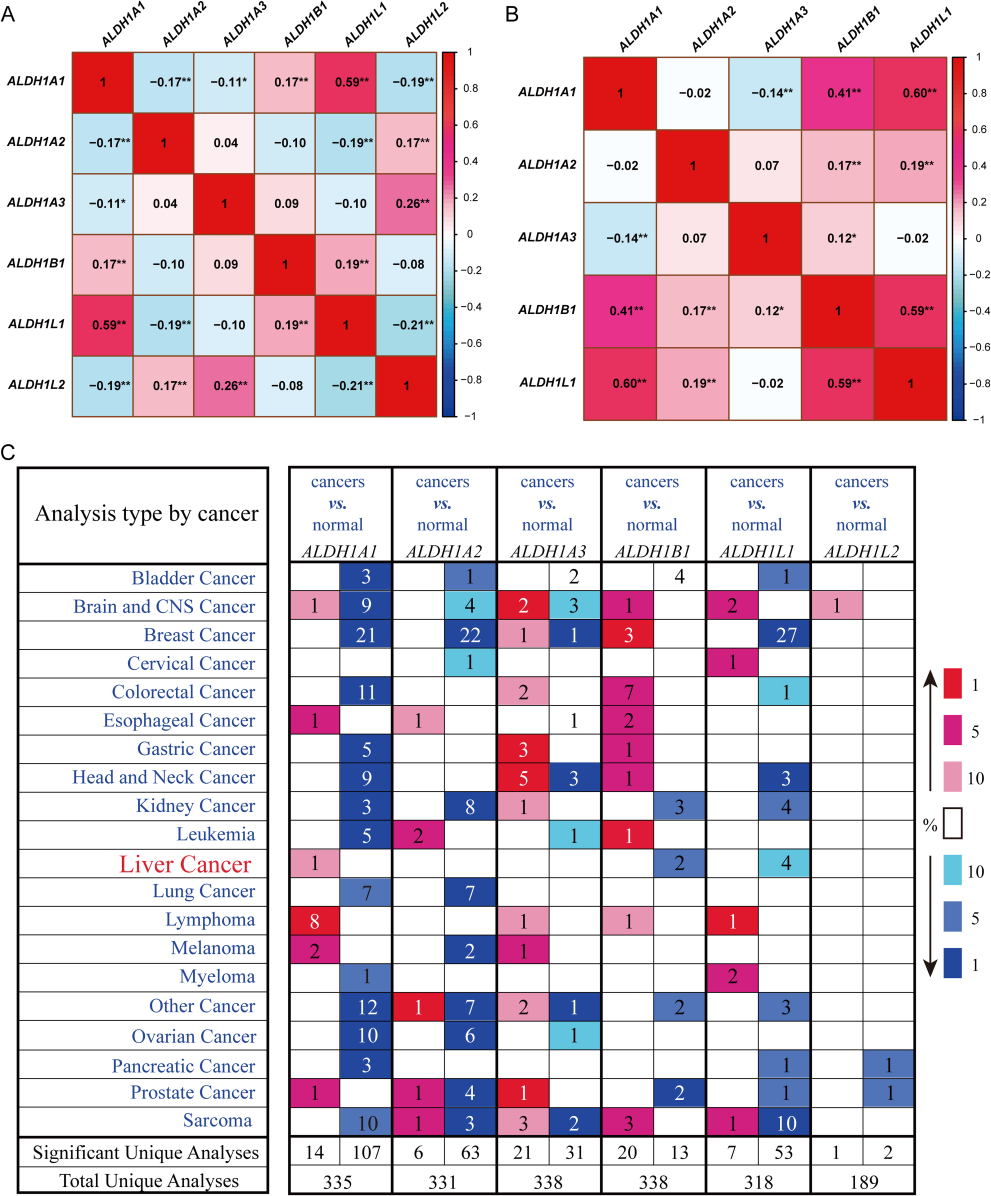
**Correlation analyses of *ALDH1* isoform mRNA expressions from the OncoLnc (A) and GEO databases (B, accession: GSE14520)**. The figure shows the association of mRNA expressions of aldehyde dehydrogenase1 (ALDH1) isoforms with a positive correlation (red) or negative correlation (blue). The numbers of cells with statistically significant correlation coefficients are denoted by ^*^P < 0.05 and ^**^P < 0.01. (C) *ALDH1* isoform mRNA expressions in different tumor types. The figure shows the number of datasets with statistically significant mRNA overexpression (red) or downregulation (blue) of the target gene (cancer *vs*. normal tissue). The *P* value threshold is 0.01. The number of datasets that met the threshold set for each experiment and the cancer types are shown. Genes were ranked by the percentile of the target genes in the top of all genes as measured in each experiment.The best gene rank percentile are denoted in color.

With the cutoff value set at the median or quartile, we determined that *ALDH1B1* was the only isoform associated with the clinical outcomes of 360 liver cancer patients in the OncoLnc database (Fig 1). There were no significant finding from analyses of the other five *ALDH1* isoforms. The high expression of *ALDH1B1* correlated with a favorable clinical outcome in liver cancer patients (log–rank, *P* = 0.0237). The baseline data of the 360 liver cancer patients are reported in S2 Table. Moreover, Tumor, Node, Metastasis (TNM) stage and associated with clinical outcomes of liver cancer patients are also reported in S2 Table. The univariate Cox proportional hazard regression model revealed a lower hazard risk in the liver cancer patients with high *ALDH1B1* expression levels (S3 Table). To further define the positive relationships among the ALDH1 isoforms, the expression levels of mRNAs were analyzed and the correlations determined using the OncLnc database. As shown in Fig 2A, we found that *ALDH1A1*, *ALDH1B1*, and *ALDH1L1* had positive relationships.

Furthermore, we downloaded the clinical data of 445 HCC and paramalignant samples with HBV infection in the GEO database. Clinicopathological characteristics of HBV-related HCC patients were shown in S4 Table (only 212 samples have entries for baseline data). Most characteristics were associated with prognosis of HBV-related HCC patients, except for age and alanine aminotransferase level. A correlation with the ALDH1 family was detected, except for *ALDH1L2*, which was not found in the GSE14520 (i.e., probably did not contain the probe for *ALDH1L2*). The results also demonstrated that *ALDH1A1*, *ALDH1B1*, and *ALDH1L1* were positively related (Fig 2B). Oncomine analyses of six ALDH1 family submembers in cancer *vs*. normal samples showed that *ALDH1A1* was significantly upregulated in liver cancer in the different datasets. In contrast, downregulation of *ALDH1B1* and *ALDH1L1* was found in liver cancer compared with normal tissues (Fig 2C). In normal tissues, the relatively higher expression levels of *ALDH1A1*, *ALDH1B1*, and *ALDH1L1* were analyzed using the GTEx database (S1 Fig). Considering these results, we speculated that *ALDH1A1/B1/L1* expression levels contributed to ALDH1 activity.

**Fig 3:**
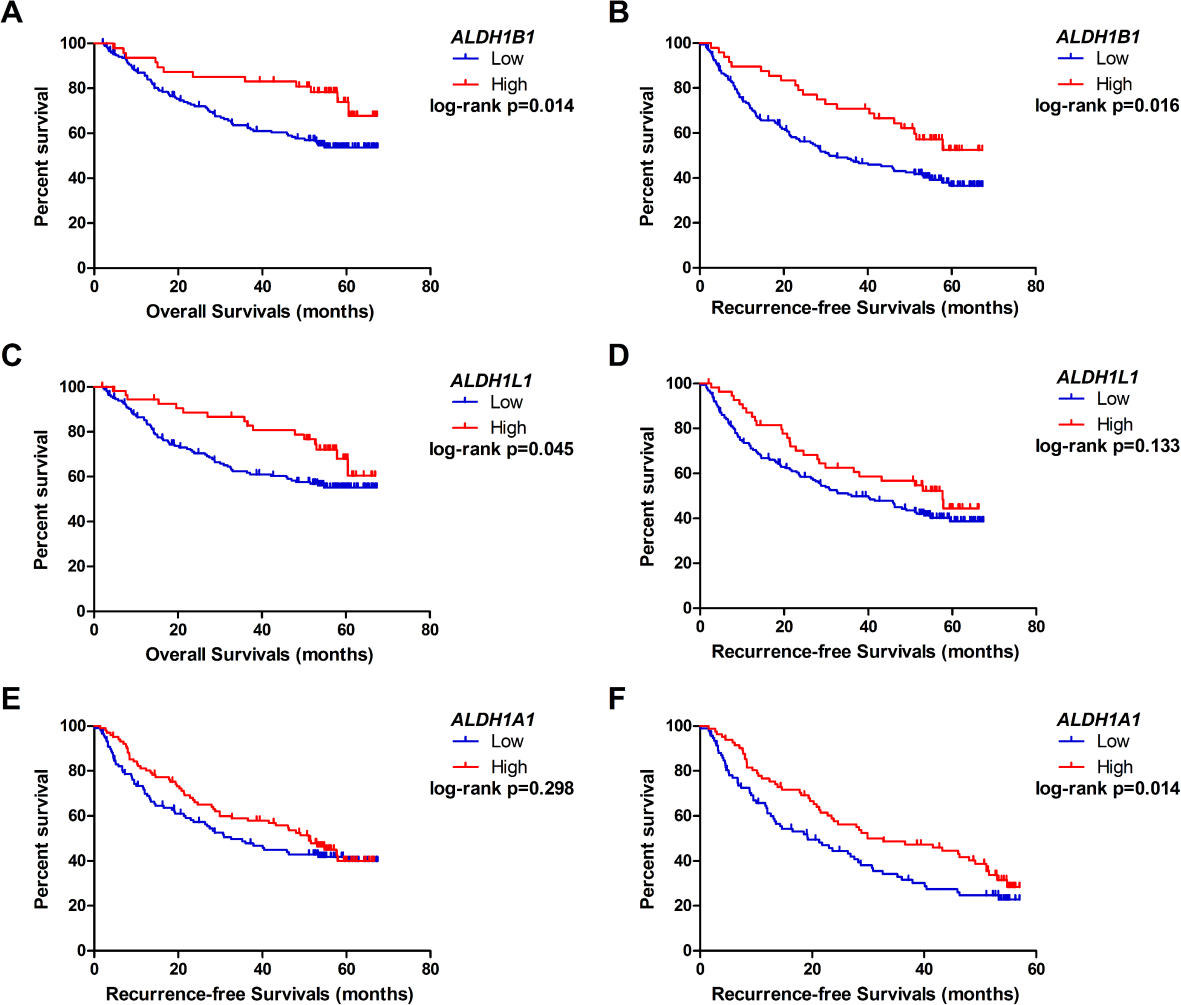
The prognostic value of *ALDH1A1/B1/L1* mRNA expression in HBV-related HCC patients [data from the GEO database (accession: GSE14520)]. A and C: The Kaplan–Meier survival analyses of aldehyde dehydrogenase (*ALDH*)*1B1* and *ALDH1L1* expression of the overall survival time of HBV-related HCC patients. B and D: The Kaplan–Meier survival analyses of *ALDH1B1* and *ALDH1L1* expression in recurrence–free survival time of HBV-related HCC patients. E and F: The Kaplan–Meier survival analyses of *ALDH1A1* expression in recurrence–free survival time and 57–month recurrence–free survival time of HBV related HCC patients, respectively. HCC = hepatocellular carcinoma.

Moreover, survival analyses have shown that high expression of *ALDH1B1* in 212 HBV-related HCC patients had a favorable outcome in terms of overall survival (OS) (Fig 3A) and recurrence–free survival (RFS) (Fig 3B). After adjusting for age and sex, high *ALDH1B1* expression had a lower risk in OS and RFS of the HBV–HCC patients (HR_OS_ = 0.46; HR_RFS_ = 0.53, respectively; Table 1). Likewise, high *ALDH1L1* mRNA expression was associated with a lower hazard in clinical outcomes of HBV–HCC patients (HR_OS_ = 0.54; HR_RFS_ = 0.65, respectively; Table 1). Using Kaplan–Meier survival analyses, high *ALDH1L1*-expressing patients had a longer OS time (Fig 3C), whereas there was no statistical difference between the two groups in RFS (Fig 3D). Although we did not find that *ALDH1A1* expression was associated with long–term clinical outcome (Fig 3E), high *ALDH1A1* mRNA expression was significantly associated with a 57–month RFS (Fig 3F) and had a lower hazard risk (HR_RFS_ = 0.67; Table 1). However, there was no statistical significance in the associations of *ALDH1A2/A3* expression levels with clinical outcomes of HBV-related HCC patients.

**Table 1:** Association between *ALDH1A1/B1/L1* mRNA expression and clinical outcomes in HBV-related HCC patients.

			OS				RFS			
Gene		number	MST	*P* value	HR ^a^	95%CI	MRT	*P* value	HR ^a^	95%CI
*ALDH1A1*	Low	119	>67.4	0.230	Ref.		32.6	0.298	Ref.	
	High	102	>67.0		0.75	0.49–1.16	51.1		0.78	0.55–1.13
*ALDH1A2*	Low	138	>67.4	0.549	Ref.		45.9	0.970	Ref.	
	High	83	>66.6		0.85	0.55–1.33	41.6		0.98	0.68–1.41
*ALDH1A3*	Low	153	>67.4	0.184	Ref.		45.9	0.927	Ref.	
	High	68	>67.3		0.73	0.44–1.20	40.1		1.02	0.69–1.51
*ALDH1B1*	Low	173	>67.4	**0.014**	Ref.		30.9	**0.016**	Ref.	
	High	48	>67.3		**0.46**	**0.25–0.84**	>67.3		**0.53**	**0.33–0.86**
*ALDH1L1*	Low	165	>67.4	**0.045**	Ref.		36.0	0.133	Ref.	
	High	56	>67.0		**0.54**	**0.31–0.94**	57.7		**0.65**	**0.42–0.996**
*ALDH1A1*^b^	Low	85	32.6	0.093	Ref.		19.2	**0.039**	Ref.	
	High	71	52.7		0.69	0.44–1.07	29.9		**0.67**	**0.47–0.97**

Note

^a^ HR adjusted for age and sex for the Cox proportional hazard model.

^b^ The association of aldehyde dehydrogenase (ALDH) 1A1 expression with the 57-month survival time and 57-month recurrence–free survival time. The bold terms are statistically significant.

**Abbreviations**: OS, overall survival; RFS, recurrence–free survival; MST, median survival time; MRT, median recurrence time; HR, hazard ratio; 95% CI, 95% confidence intervals; Ref., reference.

**Fig 4:**
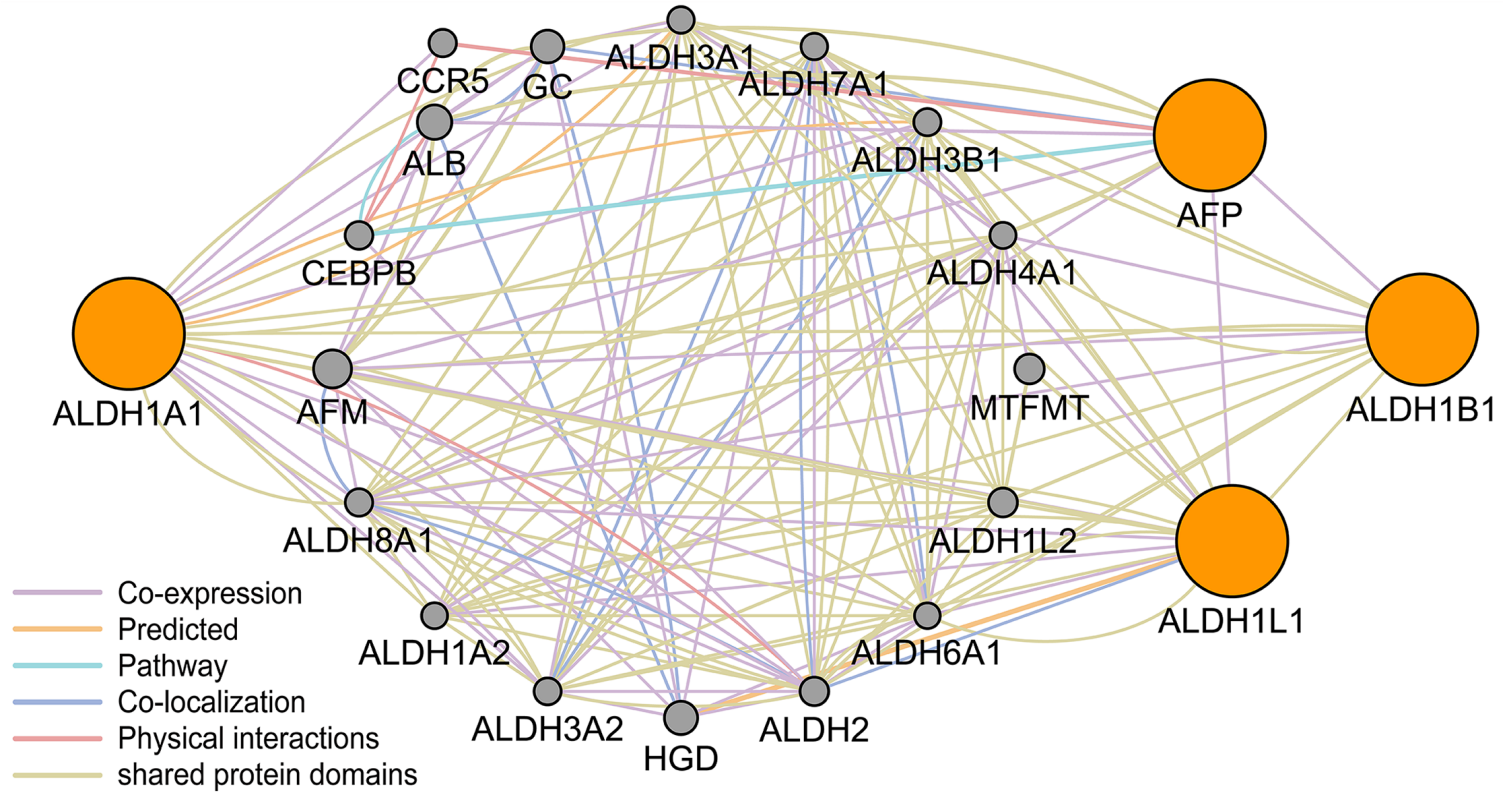
The gene pathway analysis between *ALDH1A1/B1/L1* and *AFP* using GeneMANIA software. ALDH = aldehyde dehydrogenase; AFP = alpha–fetoprotein.

We also performed logistical regression analysis adjusted for age and sex to evaluate the relationship between *ALDH1A1/B1/L1* expression and clinical features of HBV-related HCC patients (Table 2). The results demonstrated that high *ALDH1B1*-expression was a protective factor for multiple nodules (OR = 0.28, 95% CI = 0.10–0.85, *P* = 0.024). In addition, lower expression of *ALDH1B1/L1* was associated with high serum alpha–fetoprotein (AFP) levels (≥ 300 ng/mL) (OR = 0.22 and 0.39, respectively). We performed pathway analysis using GeneMANIA software, and we found, upon co–expression, that *AFP* was associated with *ALDH1B1/L1* (Fig 4).

**Table 2:** Association between *ALDH1A1/B1/L1* mRNA expression and the tumor biological features in HBV-related HCC patients.

Variables		*ALDH1A1*				*ALDH1B1*				*ALDH1L1*			
		Low	High	OR (95%CI)	*P* value	Low	High	OR (95%CI)	*P* value	Low	High	OR(95%CI)	*P* value
Tumor size	<5	70	70	Ref.		108	32	Ref.		101	39	Ref.	
(cm)	≥5	49	31	0.61 (0.34–1.07)	0.085	65	15	0.75 (0.38–1.50)	0.418	64	16	0.59 (0.30–1.16)	0.126
Nodule	single	92	84	Ref.		132	44	Ref.		129	47	Ref.	
	multiple	27	18	0.76 (0.39–1.49)	0.427	41	4	**0.28 (0.10–0.85)**	**0.024**	36	9	0.74 (0.33–1.69)	0.476
Cirrhosis	no	13	5	Ref.		14	4	Ref.		16	2	Ref.	
	yes	106	97	2.20 (0.75–6.48)	0.152	159	44	0.90 (0.28–2.94)	0.866	149	54	2.41 (0.53–11.03)	0.257
BCLC	0 or A	87	81	Ref.		132	36	Ref.		126	42	Ref.	
	B or C	31	20	0.72 (0.38–1.36)	0.308	39	12	1.16 (0.55–2.54)	0.698	38	13	1.12 (0.54–2.34)	0.757
AFP (ng/mL)	<300	87	81	Ref.		124	44	Ref.		120	48	Ref.	
	≥300	31	20	0.69 (0.36–1.32)	0.262	47	4	**0.22 (0.07–0.66)**	**0.007**	44	7	**0.39 (0.16–0.93)**	**0.033**

**Note**: OR adjusted age and sex for logistic regression, the bold terms are statistically significant. **Abbreviations**: OR, odds ratio; 95% CI, 95% confidence intervals; Ref, reference; BCLC, Barcelona Clinic Liver Cancer; AFP, alpha–fetoprotein; ALDH, aldehyde dehydrogenase; HCC, hepatocellular carcinoma.

## Discussion

Using online data resources, we showed that the expression of ALDH1 family members in normal tissues was significantly different from that in liver cancer tissues. High *ALDH1B1* expression was found to play a protective role in liver cancer patients. Moreover, HBV-related HCC patients who expressed high levels of *ALDH1L1* had a better clinical outcomes than expressed in other *ALDH1* isoforms. In addition, high *ALDH1A1* expression was associated with a 57–month RFS for HBV-related HCC patients. Multiple nodules and high serum AFP levels were considered risk factors for *ALDH1B1* expression. Furthermore, high serum AFP level may contribute to lower *ALDH1L1* expression. Therefore, they could potentially serve as diagnostic and prognostic markers as well as potential drug targets.

Epidemiological studies have unequivocally identified chronic alcohol consumption as an important risk factor for the development of various types of cancers [22]. Ethanol is absorbed by the small intestine and then metabolized, primarily by the liver. Ethanol is oxidized by cytosolic alcohol dehydrogenase (ADH) to form acetaldehyde, which is subsequently oxidized by mitochondrial aldehyde dehydrogenases, mainly ALDH2, to produce acetate [23]. Among the known human ALDHs, cytosolic ALDH1A1 has been shown to play a major role in acetaldehyde oxidation and elimination [24]. In a rodent study, cytosolic ALDH1A1 has been shown to be involved in acetaldehyde metabolism and alcohol preference [25]. A study using crude lysate from Huh7 hepatoma cells reported that ALDH1B1 contributed to the oxidation of short-chain aldehydes including acetaldehyde and propionaldehyde, which suggested a role for ALDH1B1 in ethanol metabolism [26]. Stagos et al. [27] observed the punctate positive staining of human ALDH1B1 in the human liver and pancreas using immunohistochemical staining. As Stagos et al. found a similarly higher level of *ALDH1B1* and *ALDH2* mRNAs in mouse liver relative to other tissues examined, these results further suggested an important role for the two mitochondrial ALDHs in ethanol metabolism. Furthermore, the ALDH1L1 enzyme appeared to be a chief regulator of cellular metabolism, as it is strongly downregulated under certain physiological and pathological conditions [28, 29], while its upregulation can produce drastic antiproliferative effects [28]. Hwang et al. [30] proposed a hypothesis that upregulating ALDH1L1 expression in order to decrease acetaldehyde concentrations and promote DNA stability would thereby decrease cancer susceptibility.

Several markers have been identified for the selection of human (cancer) stem cells, of which ALDH1 is among the most widely studied. ALDH1 consists of detoxifying enzymes that are responsible for the oxidation of retinaldehydes to retinoids. Previous reports of the liver evaluated by flow cytometry suggested that high ALDH activity could be a marker of liver progenitor cells in normal liver [31] and cancer stem cells in HCC cases [32]. In humans, ALDH1 has six subfamily members, which exhibit > 60% amino acid identity, with ALDH1A1 as the predominant isoform [33]. Previous studies showed that upregulation of *ALDH1A1* expression was associated with enhanced invasiveness in acute myeloid leukemia[34], nasopharyngeal carcinoma [35], bladder cancer [36], and pancreatic cancer [37]. Moreb et al. [38] reported that disrupting *ALDH1A1* using small interfering (si) RNAs in lung cancer cell lines reduced neoplastic activity. However, we found that high *ALDH1A1* expression was associated with a better 57–month RFS in HBV-related HCC patients. The results were consistent with those of Suzuki et al.[39], which suggested that high ALDH1A1 was a favorable prognostic factor in RFS of HCC patients. Compared to the normal tissues in the Oncomine database, ALDH1A1 expression was upregulated in HCC tissues. The result came from Mas et al. [40] study, enrolling 88 distinct patients with hepatitis C viral (HCV) infection (41 HCV-cirrhotic tissues,17 HCV-cirrhotic tissues from patients with HCC, and 47 HCV-HCC tissues). Differentially expressed genes were studied by use of high-density oligonucleotide arrays, including hybridized to HG-U133A and HG-U133A 2.0 GeneChips. We therefore speculated that *ALDH1A1* expression was involved in cancer heterogeneity and the differences in prognoses for different virus infection backgrounds and types of cancer.

*ALDH1B1*, a mitochondrial ALDH, is another promising good prognostic marker for cancer, sharing 65% homology in peptide sequence with cytosolic *ALDH1A1* in humans [41]. *ALH1B1* catalytically metabolizes a wide range of aldehyde substrates, including acetaldehyde and products of lipid peroxidation, and is activated in ethanol metabolism [27]. In our gene ontology annotation studies, the main functions of *ALDH1B1* were aldehyde dehydrogenase activity and oxidoreductase activity. Singh et al. [42] suggested that *ALDH1B1*, a significant contributor to ALDH activity as measured by the Aldefluor^™^ assay, could promote tumor formation by modulating the Wnt/β–catenin, Notch, and PI3K/Akt signaling pathways. However, *ALDH1B1* is a relatively uncharacterized member of the ALDH1 superfamily in the context of HCC. In the present study, we found that high *ALDH1B1* expression in HCC patients had a favorable clinical survival prediction for OS and RFS. From the Oncomine database, we found that ALDH1B1 expression was downregulated in liver cancer tissues compared to that of the normal tissues, which suggested *ALDH1B1* as a potential tumor suppressor gene. Furthermore, multiple nodules and high AFP expression could lead to the downregulation of *ALDH1B1* expression. A relationship of co–repression between *AFP* and *ALDH1B1*was identified via the GeneMANIA software. Although *ALDH1B1* could be a potential prognostic marker for HCC patients, the mechanism is still undetermined.

*ALDH1L1*, another member of the ALDH gene superfamily, is also called FDH [10–formyl tetrahydrofolate (THF) dehydrogenase] and is involved in nervous system development and reduced proliferation. These findings imply that *ALDH1L1* may have tumor suppressor properties [43]. Oleinik et al. found that *ALDH1L1* suppressed cancer cell proliferation by depleting intracellular 10–formyl THF, which is essential for *de novo* purine biosynthesis. This effect could be reversed by excess extracellular folate [44]. Future investigations regarding the critical role of *ALDH1L1* in cancer cell survival and induction of folate stress will provide important insights into the malignant process. Furthermore, these studies could link deregulation of key metabolic pathways to cancer, as well as establish new targets for diagnostics of the malignant transformation process. Microarray data showed that mRNA levels of *ALDH1L1* were remarkably reduced in HCC [45]. A previous study [46] assessed *ALDH1L1* expression in HCC tissues using real–time quantitative RT–PCR, western blotting, and immunohistochemistry staining, and further findings showed that low expression of *ALDH1L1* in HCC was significantly associated with pathology grade, hepatitis B surface antigen status, and serum AFP. The studies also suggested that decreased expression of *ALDH1L1* was associated with a poor prognosis in HCC patients. Using gene pathway analysis, we found that *AFP* was associated with the ALDH1 family. Concerning the results of our study, high *ALDH1L1* expression was associated with prognosis and serum AFP levels, which was consistent with previous studies. Thus, *ALDH1L1* may be a novel prognostic marker for HCC, but the specific mechanism remains unclear.

Several limitations of this study warrant discussion. First, our sample size was limited and a small fraction of the clinical data was missing. As such, additional studies with larger sample sizes will be needed to clarify our results. Second, the subjects evaluation collected in this study included background and type of tumor, which may introduce background heterogeneity and may also represent a major limitation of the study. Third, we specifically used median mRNA expression to evaluate the prognostic value in HBV-related HCC patients, the appropriate cut-off value of ALDH1 isoform mRNA expression for HCC patients remains unclear.

In conclusion, our findings demonstrated that *ALDH1A1*, *ALDH1B1*, and *ALDH1L1* contributed to the *ALDH1* family activity. The expressions of *ALDH1B1* and *ALDH1L1* were associated with clinical features and survival of HBV-related HCC patients. Moreover, high *ALDH1A1* expression in HBV-related HCC patients had a favorable 57–month RFS time. The results provided a better understanding of the heterogeneity and complexity of HCC at the molecular level, and further provided a foundation of more accurate prognoses and the development of new treatment targets.

## Supporting information

S1 Fig The mRNA expression of *ALDH1A1/B1/L1* in normal tissues as analyzed from the GTEx database. Red frame is normal liver tissue. ALDH = aldehyde dehydrogenase.
(TIF)

S1 Table DAVID gene ontology terms for *ALDH1* isoforms. (DOCX)

S2 Table Clinicopathological characteristics of liver cancer cases based on TCGA database from OncoLnc website. (DOCX)

S3 Table Association between ALDH1 isoforms and clinical outcomes in liver cancer patients. (DOCX)

S4 Table Clinicopathological characteristics of HBV-related HCC cases in GSE14520 from GEO database. (DOCX)
