# Supplementary figures and images for: Aldehyde dehydrogenase 1 (ALDH1) isoform expression and potential clinical implications in hepatocellular carcinoma
Source: PLoS One. 2017 Aug 8;12(8):e0182208. doi: 10.1371/journal.pone.0182208 (PMC5549701; doi:10.1371/journal.pone.0182208)

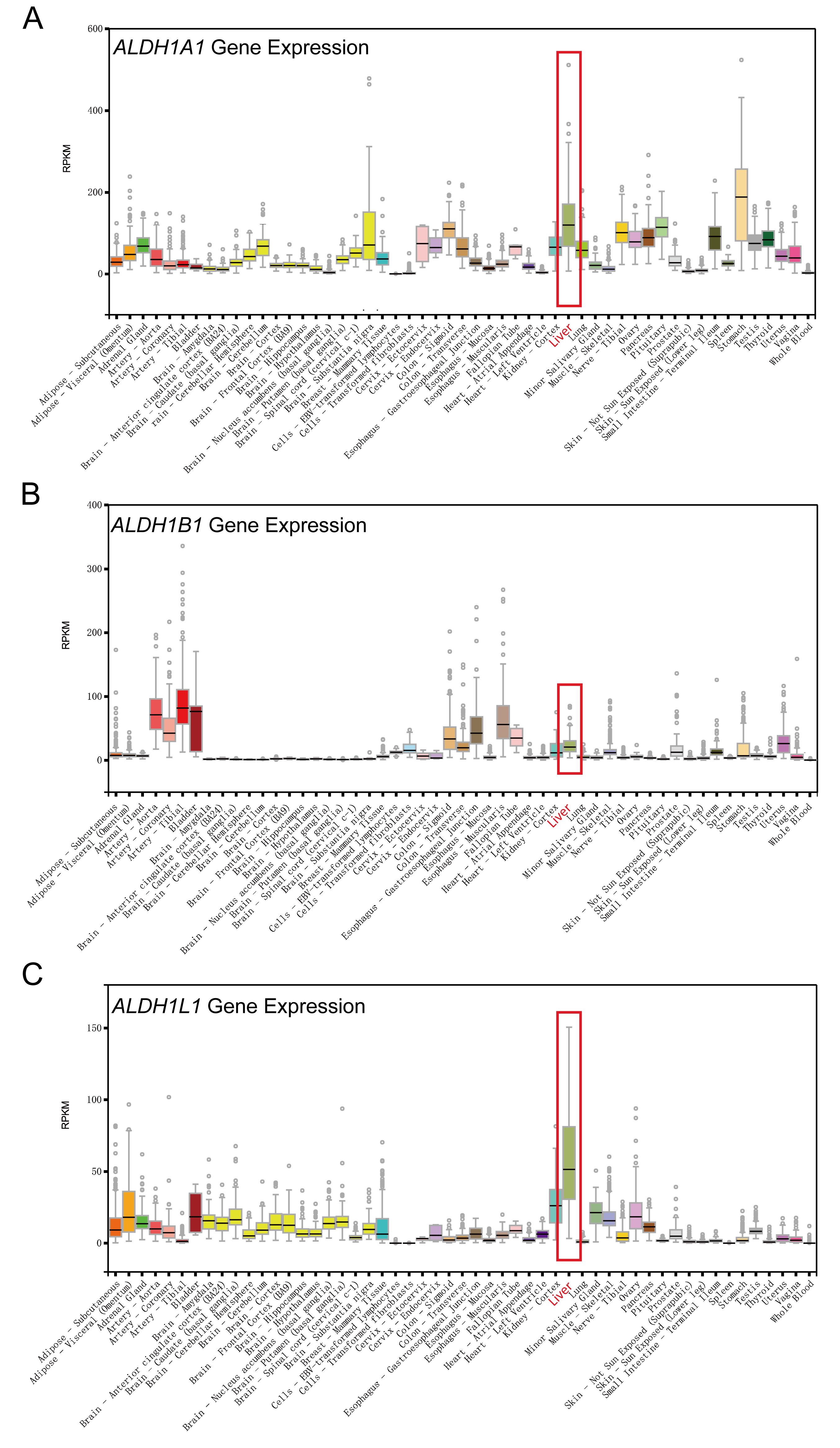

Supplement: S1 Fig — Red frame is normal liver tissue. ALDH = aldehyde dehydrogenase. (TIF) [file pone.0182208.s001.tif]
