# Supplementary material for: Aldehyde dehydrogenase 1 (ALDH1) isoform expression and potential clinical implications in hepatocellular carcinoma
Source: PLoS One. 2017 Aug 8;12(8):e0182208. doi: 10.1371/journal.pone.0182208 (PMC5549701; doi:10.1371/journal.pone.0182208)
Supplement: S1 Table — (DOCX) [file pone.0182208.s002.docx]

**S1 Table. DAVID gene ontology terms for *ALDH1* isoforms.**

| Gene ontology term | *P* value | Genes | Bonferroni | Benjamini |
| --- | --- | --- | --- | --- |
| aldehyde dehydrogenase (ALDH) (NAD) activity | 5.42E–16 | *ALDH1A1*, *ALDH1A2*, *ALDH1L1*, *ALDH1B1*, *ALDH1A3*, *ALDH1L2* | 9.44E–15 | 9.44E–15 |
| oxidoreductase activity, acting on the aldehyde or oxo group of donors, NAD or NADP as an acceptor | 5.73E–12 | *ALDH1A1*, *ALDH1A2*, *ALDH1L1*, *ALDH1A3*, *ALDH1L2* | 9.73E–11 | 4.87E–11 |
| oxidoreductase activity | 9.47E–08 | *ALDH1A1*, *ALDH1A2*, *ALDH1L1*, *ALDH1B1*, *ALDH1A3* | 1.61E–06 | 5.37E–07 |
| retinal dehydrogenase activity | 1.47E–06 | *ALDH1A1*, *ALDH1A2*, *ALDH1A3* | 2.50E–05 | 6.26E–06 |
| oxidation–reduction process | 7.43E–06 | *ALDH1A1*, *ALDH1A2*, *ALDH1L1*, *ALDH1A3*, *ALDH1L2* | 4.39E–04 | 4.39E–04 |
| retinol metabolic process | 3.08E–05 | *ALDH1A1*, *ALDH1A2*, *ALDH1A3* | 1.81E–03 | 9.07E–04 |
| formyl tetrahydrofolate dehydrogenase activity | 5.92E–04 | *ALDH1L1*, *ALDH1L2* | 1.00E–02 | 2.01E–03 |
| 10–formyl tetrahydrofolate catabolic process | 5.95E–04 | *ALDH1L1*, *ALDH1L2* | 3.45E–02 | 1.16E–02 |
| hydroxymethyl–, formyl– and related transferase activity | 8.88E–04 | *ALDH1L1*, *ALDH1L2* | 1.50E–02 | 2.51E–03 |
| 4–trimethylammonium butyraldehyde dehydrogenase activity | 1.18E–03 | *ALDH1L1*, *ALDH1L2* | 1.99E–02 | 2.87E–03 |

**Note**: DAVID = Database for Annotation, Visualization and Integrated Discovery; NAD = Nicotinamide Adenine Dinucleotide**;** NADP = Nicotinamide Adenine Dinucleotide Phosphate.
