# Supplementary material for: Aldehyde dehydrogenase 1 (ALDH1) isoform expression and potential clinical implications in hepatocellular carcinoma
Source: PLoS One. 2017 Aug 8;12(8):e0182208. doi: 10.1371/journal.pone.0182208 (PMC5549701; doi:10.1371/journal.pone.0182208)
Supplement: S2 Table — (DOCX) [file pone.0182208.s003.docx]

**S2 Table. Clinicopathological characteristics of liver cancer cases of OncoLnc website from TCGA database.**

| Variable | | ALDH1A1 | | χ^2^ | *p* | ALDH1A2 | | χ^2^ | *p* | ALDH1A3 | | χ^2^ | *p* | ALDH1B1 | | χ^2^ | *p* | ALDH1L1 | | χ^2^ | *p* | ALDH1L2 | | χ^2^ | *p* | MST | ^a^ *p* |
| --- | --- | --- | --- | --- | --- | --- | --- | --- | --- | --- | --- | --- | --- | --- | --- | --- | --- | --- | --- | --- | --- | --- | --- | --- | --- | --- | --- |
|  |  | low | high |  |  | low | high |  |  | low | high |  |  | low | high |  |  | low | high |  |  | low | high |  |  |  |  |
|  |  | (n=180) | (n=180) |  |  | (n=180) | (n=180) |  |  | (n=180) | (n=180) |  |  | (n=270) | (n=90) |  |  | (n=180) | (n=180) |  |  | (n=180) | (n=180) |  |  | (months) |  |
| Rice | Asian | 80 | 75 | 1.05 | 0.592 | 81 | 74 | 0.87 | 0.647 | 85 | 70 | 3.90 | 0.143 | 116 | 39 | 3.99 | 0.136 | 80 | 75 | 2.23 | 0.329 | 91 | 64 | **11.34** | **0.003** | >88.7 | 0.398 |
|  | Black | 7 | 11 |  |  | 8 | 10 |  |  | 10 | 8 |  |  | 17 | 1 |  |  | 6 | 12 |  |  | 11 | 7 |  |  | 37.1 |  |
|  | White | 89 | 89 |  |  | 85 | 93 |  |  | 79 | 99 |  |  | 130 | 48 |  |  | 91 | 87 |  |  | 73 | 105 |  |  | 45.1 |  |
| Age | ≤60 yr | 93 | 80 | 1.88 | 0.170 | 81 | 92 | 1.35 | 0.246 | 79 | 94 | 2.50 | 0.114 | 137 | 36 | 3.12 | 0.077 | 103 | 70 | **12.12** | **<0.001** | 83 | 90 | 0.55 | 0.460 | 81.7 | 0.274 |
|  | >60 yr | 87 | 100 |  |  | 99 | 88 |  |  | 101 | 86 |  |  | 133 | 54 |  |  | 77 | 110 |  |  | 97 | 90 |  |  | 52.3 |  |
| Gender | Male | 101 | 143 | **22.44** | **<0.001** | 134 | 110 | **7.33** | **0.007** | 126 | 118 | 0.81 | 0.367 | 181 | 63 | 0.27 | 0.602 | 98 | 146 | **29.31** | **<0.001** | 122 | 122 | <0.01 | 1.000 | 80.2 | 0.310 |
|  | Female | 79 | 37 |  |  | 46 | 70 |  |  | 54 | 62 |  |  | 89 | 27 |  |  | 82 | 34 |  |  | 58 | 58 |  |  | 50.3 |  |
| TNM stage | I | 76 | 93 | 5.85 | 0.054 | 78 | 91 | 1.16 | 0.561 | 81 | 88 | 1.71 | 0.426 | 121 | 48 | 5.20 | 0.074 | 74 | 95 | 5.14 | 0.077 | 92 | 77 | 1.85 | 0.397 | **81.7** | **<0.001** |
|  | II | 42 | 41 |  |  | 42 | 41 |  |  | 47 | 36 |  |  | 70 | 13 |  |  | 42 | 41 |  |  | 39 | 44 |  |  | **105.1** |  |
|  | ≥III | 53 | 34 |  |  | 46 | 41 |  |  | 45 | 42 |  |  | 63 | 24 |  |  | 51 | 36 |  |  | 41 | 46 |  |  | **24.8** |  |
| Grade | G1 | 24 | 31 | 3.35 | 0.341 | 28 | 27 | 1.17 | 0.759 | 25 | 30 | 3.70 | 0.259 | 34 | 21 | **8.10** | **0.044** | 22 | 33 | **8.92** | **0.030** | 26 | 29 | **8.37** | **0.039** | 68.3 | 0.748 |
|  | G2 | 84 | 91 |  |  | 84 | 91 |  |  | 82 | 93 |  |  | 132 | 43 |  |  | 80 | 95 |  |  | 79 | 96 |  |  | 54.4 |  |
|  | G3 | 64 | 54 |  |  | 63 | 55 |  |  | 65 | 53 |  |  | 96 | 22 |  |  | 70 | 48 |  |  | 65 | 53 |  |  | 52.3 |  |
|  | G4 | 8 | 4 |  |  | 5 | 7 |  |  | 8 | 4 |  |  | 8 | 4 |  |  | 8 | 4 |  |  | 10 | 2 |  |  | >40.1 |  |

**Note**: ^a^ *P* is for univariate survival analysis. The bold terms are statistical significance.

**Abbreviations**: MST, median survival time; TNM: Tumor, Node, Metastasis.
