# Supplementary material for: Aldehyde dehydrogenase 1 (ALDH1) isoform expression and potential clinical implications in hepatocellular carcinoma
Source: PLoS One. 2017 Aug 8;12(8):e0182208. doi: 10.1371/journal.pone.0182208 (PMC5549701; doi:10.1371/journal.pone.0182208)
Supplement: S3 Table — (DOCX) [file pone.0182208.s004.docx]

**S3 Table. Association between ALDH1 isoforms and clinical outcomes in liver cancer patients.**

| Gene expression | | number | MST (days) | *P* value | HR^a^ | 95%CI |
| --- | --- | --- | --- | --- | --- | --- |
| *ALDH1A1* | low | 180 | 1791 | 0.96 | Ref. |  |
|  | high | 180 | 1694 |  | 1.01 | 0.71–1.43 |
| *ALDH1A2* | low | 180 | 1490 | 0.32 | Ref. |  |
|  | high | 180 | 2116 |  | 0.84 | 0.59–1.19 |
| *ALDH1A3* | low | 180 | 1423 | 0.70 | Ref. |  |
|  | high | 180 | 2116 |  | 0.72 | 0.51–1.03 |
| *ALDH1B1* | low | 270 | 1560 | **0.02** | Ref. |  |
|  | high | 90 | 2131 |  | **0.59** | **0.37–0.94** |
| *ALDH1L1* | low | 180 | 1490 | 0.08 | Ref. |  |
|  | high | 180 | 2456 |  | 0.73 | 0.51–1.04 |
| *ALDH1L2* | low | 180 | 1694 | 0.34 | Ref. |  |
|  | high | 180 | 1791 |  | 0.84 | 0.59–1.20 |

**Note**: ^a^HR for univariate Cox proportional hazard model. The bold terms are statistically significant.

**Abbreviations**: MST, median survival time; HR, hazard ratio; 95% CI (confidence interval); ALDH, aldehyde dehydrogenase; Ref., reference.
