# Supplementary material for: Aldehyde dehydrogenase 1 (ALDH1) isoform expression and potential clinical implications in hepatocellular carcinoma
Source: PLoS One. 2017 Aug 8;12(8):e0182208. doi: 10.1371/journal.pone.0182208 (PMC5549701; doi:10.1371/journal.pone.0182208)
Supplement: S4 Table — (DOCX) [file pone.0182208.s005.docx]

**S4 Table. Clinicopathological characteristics of HBV-related HCC cases of GSE14520 from GEO database.**

| Variable | | ALDH1A1 | | χ^2^ | *p* | ALDH1A2 | | χ^2^ | *p* | ALDH1A3 | | χ^2^ | *p* | ALDH1B1 | | χ^2^ | *p* | ALDH1L1 | | χ^2^ | *p* | MST | ^a^ *p* | MRT | ^a^ *p* |
| --- | --- | --- | --- | --- | --- | --- | --- | --- | --- | --- | --- | --- | --- | --- | --- | --- | --- | --- | --- | --- | --- | --- | --- | --- | --- |
|  |  | low | high |  |  | low | high |  |  | low | high |  |  | low | high |  |  | low | high |  |  |  |  |  |  |
|  |  | (n=106) | (n=106) |  |  | (n=106) | (n=106) |  |  | (n=106) | (n=106) |  |  | (n=106) | (n=106) |  |  | (n=106) | (n=106) |  |  | (months) |  | (months) |  |
| Age | ≤50 yr | 57 | 52 | 0.47 | 0.459 | 53 | 56 | 0.17 | 0.680 | 51 | 58 | 0.93 | 0.336 | 57 | 52 | 0.47 | 0.492 | 58 | 51 | 0.93 | 0.336 | >67.4 | 0.813 | 51.6 | 0.236 |
|  | >50 yr | 49 | 54 |  |  | 53 | 50 |  |  | 55 | 48 |  |  | 49 | 54 |  |  | 48 | 55 |  |  | >67.1 |  | 35.2 |  |
| Gender | Male | 93 | 90 | 0.36 | 0.549 | 90 | 93 | 0.36 | 0.549 | 91 | 92 | 0.04 | 0.842 | 94 | 89 | 0.99 | 0.318 | 89 | 94 | 0.99 | 0.318 | >67.4 | 0.148 | **40.1** | **0.018** |
|  | Female | 13 | 16 |  |  | 16 | 13 |  |  | 15 | 14 |  |  | 12 | 17 |  |  | 17 | 12 |  |  | >67.1 |  | **>67.1** |  |
| ALT | <50 U/ml | 67 | 57 | 1.94 | 0.163 | 63 | 61 | 0.08 | 0.780 | 66 | 58 | 1.24 | 0.265 | 59 | 65 | 0.70 | 0.403 | 61 | 63 | 0.08 | 0.780 | >67.3 | 0.684 | 53 | 0.246 |
|  | ≥50 U/ml | 39 | 49 |  |  | 43 | 45 |  |  | 40 | 48 |  |  | 47 | 41 |  |  | 45 | 43 |  |  | >67.4 |  | 40.4 |  |
| AFP | ≤300 ng/ml | 42 | 73 | **17.93** | **<0.001** | 51 | 64 | 3.55 | 0.060 | 55 | 60 | 0.60 | 0.440 | 49 | 66 | **5.96** | **0.015** | 43 | 72 | **15.65** | **<0.001** | **>67.4** | **0.047** | 48 | 0.327 |
|  | >300 ng/ml | 62 | 32 |  |  | 54 | 40 |  |  | 50 | 44 |  |  | 56 | 38 |  |  | 61 | 33 |  |  | **>67.1** |  | 35.2 |  |
| Tumor size | <5 cm | 63 | 74 | 2.82 | 0.093 | 68 | 69 | <0.01 | 0.960 | 70 | 67 | 0.12 | 0.735 | 65 | 72 | 1.22 | 0.270 | 58 | 79 | **9.76** | **0.002** | **>67.4** | **0.002** | 51.1 | 0.073 |
|  | ≥5 cm | 43 | 31 |  |  | 37 | 37 |  |  | 36 | 38 |  |  | 41 | 33 |  |  | 48 | 26 |  |  | **53.3** |  | 28.4 |  |
| Nodular | Single | 83 | 84 | 0.03 | 0.867 | 87 | 80 | 1.38 | 0.240 | 82 | 85 | 0.25 | 0.614 | 81 | 86 | 0.71 | 0.401 | 79 | 88 | 2.29 | 0.131 | >67.4 | 0.052 | 49.1 | 0.381 |
|  | Multiple | 23 | 22 |  |  | 19 | 26 |  |  | 24 | 21 |  |  | 25 | 20 |  |  | 27 | 18 |  |  | 47.9 |  | 38.7 |  |
| Cirrhosis | No | 11 | 6 | 1.60 | 0.206 | 8 | 9 | 0.06 | 0.800 | 11 | 6 | 1.60 | 0.206 | 7 | 10 | 0.58 | 0.448 | 10 | 7 | 0.58 | 0.448 | **>67.3** | **0.025** | **67.3** | **0.029** |
|  | Yes | 95 | 100 |  |  | 98 | 97 |  |  | 95 | 100 |  |  | 99 | 96 |  |  | 96 | 99 |  |  | **>67.4** |  | **37.9** |  |
| TNM stage | I | 42 | 47 | 1.38 | 0.503 | 47 | 42 | 1.31 | 0.518 | 38 | 51 | 3.82 | 0.148 | 37 | 52 | **6.18** | **0.046** | 36 | 53 | **10.98** | **0.004** | **>67.4** | **<0.001** | **>67.4** | **<0.001** |
|  | II | 37 | 39 |  |  | 34 | 42 |  |  | 44 | 32 |  |  | 39 | 37 |  |  | 37 | 39 |  |  | **>67.1** |  | **28.7** |  |
|  | ≥III | 27 | 20 |  |  | 25 | 22 |  |  | 24 | 23 |  |  | 30 | 17 |  |  | 33 | 14 |  |  | **18** |  | **18** |  |
| BCLC stage | 0 | 10 | 10 | 1.10 | 0.777 | 12 | 8 | 4.43 | 0.219 | 11 | 9 | 0.72 | 0.868 | 9 | 11 | 2.54 | 0.468 | 9 | 11 | 6.02 | 0.111 | **>67.4** | **<0.001** | **>67.3** | **<0.001** |
|  | A | 69 | 74 |  |  | 69 | 74 |  |  | 71 | 72 |  |  | 68 | 75 |  |  | 65 | 78 |  |  | **>58.4** |  | **51.6** |  |
|  | B | 11 | 11 |  |  | 8 | 14 |  |  | 12 | 10 |  |  | 12 | 10 |  |  | 14 | 8 |  |  | **46.1** |  | **26.9** |  |
|  | C | 16 | 11 |  |  | 17 | 10 |  |  | 12 | 15 |  |  | 17 | 10 |  |  | 18 | 9 |  |  | **13.6** |  | **8.9** |  |

**Note**: ^a^ *P* is for univariate survival analysis. The bold terms are statistical significance.

**Abbreviations**: MST, median survival time; MRT, median recurrence time; ALT, Alanine aminotransferase; TNM: Tumor, Node, Metastasis; BCLC, Barcelona Clinic Liver Cancer; AFP, alpha–fetoprotein; ALDH, aldehyde dehydrogenase; HCC, hepatocellular carcinoma.
